# Vaginal Hysterotomy

**Published:** 1886-05

**Authors:** W. T. Collins

**Affiliations:** Grove, Texas


### VAGINAL HYSTEROTOMY.

*By W. T. Collins, M. D., Grove, Texas.*

(For Daniel's Texas Medical Journal.)

AT 9 o'clock p. m., March 3, I was called to attend Mrs. W., a primipara, aged 26 years; found her in labor; pains coming on every twenty to twenty-five minutes. I made a careful vaginal examination, but could detect no os tincæ, it being, as I supposed, high and retroverted, which I frequently find.

Watched with my patient until 3 o'clock a. m., at which time I made a second very careful examination, and found an occluded os uteri, surrounded and bridled by firm, unyielding cicatricial bands. The os was so small that I could not force the end of my finger into it.

For three more long and anxious hours I watched, hoping that nature might be able to overcome the difficulty, and relieve my unfortunate patient without surgical interference, but I was sorely disappointed;for at 6 o'clock,nine hours since labor had commenced, the os was still rigid and undilated and undilatable.

The expulsive pains for the last three hours were very frequent and hard, and the woman was troubled by incessant vomiting, which was rapidly telling on her strength. The case was now assuming a dangerous phase, and I decided that nothing but an operation would save the mother and give a chance for the life of the babe.

I informed her mother (her husband being dead) of the necessity for the operation which I was preparing to make, to which she consented.

The patient was put upon her back, the hips brought to the edge of the bed, when I sought the cicatricial os with the index fingers of my left hand. Then using the finger as a guide, I carefully introduced a bistoury to the side of the obstruction, when I succeeded in making an anterior and posterior cut in mouth of the womb, to the extent of a half inch or more. I aided the dilatation all I could with the fingers and waited another hour, when I found the child's head corrugated and now fully engaged in the superior strait. Again I used the knife, making a free left lateral incision, through a cartilaginous band. This let the head descend and labor progressed well enough until the head reached the perineum, where it hung for four more tedious hours, when the parts yielded, and labor, like everything earthly, ended.

I repeat this case (1) from its extreme rarity ; (2) because it was produced (as I think) by syphilitic ulceration of the womb. Her husband had syphilis. The mother convalesced rapidly, an d she and the babe are both doing well.

